# Polymorphisms in Genes Encoding Glutathione Transferase Pi and Glutathione Transferase Omega Influence Prostate Cancer Risk and Prognosis

**DOI:** 10.3389/fmolb.2021.620690

**Published:** 2021-04-14

**Authors:** Veljko Santric, Dejan Dragicevic, Marija Matic, Milica Djokic, Marija Pljesa-Ercegovac, Tanja Radic, Sonja Suvakov, Marina Nikitovic, Vesna Stankovic, Bogomir Milojevic, Milan Radovanovic, Zoran Dzamic, Tatjana Simic, Ana Savic-Radojevic

**Affiliations:** ^1^ Faculty of Medicine, University of Belgrade, Belgrade, Serbia; ^2^ Clinic of Urology, Clinical Center of Serbia, Belgrade, Serbia; ^3^ Institute of Medical and Clinical Biochemistry, Belgrade, Serbia; ^4^ Institute for Oncology and Radiology of Serbia, Belgrade, Serbia; ^5^ Institute of Mental Health, Belgrade, Serbia; ^6^ Serbian Academy of Sciences and Arts, Belgrade, Serbia

**Keywords:** GSTO, GSTP1, polymorphism, prostate cancer, risk, prognosis

## Abstract

Considering the pleiotropic roles of glutathione transferase (GST) omega class members in redox homeostasis, we hypothesized that polymorphisms in *GSTO1* and *GSTO2* might contribute to prostate cancer (PC) development and progression. Therefore, we performed a comprehensive analysis of *GSTO1* and *GSTO2* SNPs’ role in susceptibility to PC, as well as whether they might serve as prognostic biomarkers independently or in conjunction with other common GST polymorphisms (*GSTM1*, *GSTT1*, and *GSTP1*). Genotyping was performed in 237 PC cases and 236 age-matched controls by multiplex PCR for deletion of GST polymorphisms and quantitative PCR for SNPs. The results of this study, for the first time, demonstrated that homozygous carriers of both GSTO1*A/A and GSTO2*G/G variant genotypes are at increased risk of PC. This was further confirmed by haplotype analysis, which showed that H2 comprising both GSTO1*A and GSTO2*G variant alleles represented a high-risk combination. However, the prognostic relevance of polymorphisms in GST omega genes was not found in our cohort of PC patients. Analysis of the role of other investigated GST polymorphisms (*GSTM1*, *GSTT1*, and *GSTP1*) in terms of PC prognosis has shown shorter survival in carriers of GSTP1*T/T (*rs1138272*) genotype than in those carrying at least one referent allele. In addition, the presence of GSTP1*T/T genotype independently predicted a four-fold higher risk of overall mortality among PC patients. This study demonstrated a significant prognostic role of GST polymorphism in PC.

## Introduction

Prostate cancer (PC) is second to lung cancer in terms of the number of cancer deaths among men in the United States and European Union, representing the sixth leading cause of all cancer deaths worldwide (Bray et al., 2018). Specific characteristics of the majority of PCs, including the slow growth rate and low metastatic potential, imply the significance of recognition of high-risk patients who are candidates for aggressive therapy at the time of the first diagnosis in comparison to those who can be managed conservatively through active surveillance (Mottet et al., 2017; Siegel et al., 2019). Despite recent advances in understanding the mechanisms underlying prostate carcinogenesis, there is a constant need for novel susceptibility, progression, and prediction of biomarkers’ development.

Representing the superfamily of detoxifying enzymes with overlapping biotransformational capacities toward xenobiotics, as well as endogenous reactive oxygen species (Wu and Dong, 2012), the role of glutathione transferases (GSTs) has been extensively studied in the development and progression of different cancers (Pljesa-Ercegovac et al., 2018). In parallel to their catalytic functions, various GSTs’ regulatory roles involved in the regulation of redox homeostasis, as well as cell survival and apoptotic signaling pathways, also affirmed their functional significance (Tew and Townsend, 2012; Board and Menon, 2013). The most established epigenetic biomarker in PC is the silencing of glutathione transferase P1 (GSTP1) being recognized as a hallmark of prostate carcinogenesis (Martignano et al., 2016; Wang et al., 2017). Besides, the protective effect of *GSTP1* haplotype associated with more efficient protection against carcinogenic compounds in PC susceptibility has been suggested recently (Santric et al., 2020). Apart from its catalytic detoxifying role, *GSTP1* is involved in the process of glutathionylation, as well as regulation of redox-dependent apoptotic signaling (Pljesa-Ercegovac et al., 2018). Regarding glutathionylation, it represents the posttranslational modification of protein thiol groups by the formation of mixed disulfides with glutathione. It seems reasonable to assume that epigenetic changes in *GSTP1* expression in the course of PC development at least partially affect the process of glutathionylation, resulting in decreased protection of protein thiol groups (Zhang et al., 2019). Moreover, reversible glutathionylation/deglutathionylation has the capacity to act as a regulatory switch, modulating individual enzymes or more complex pathways of cellular metabolism and function (Board and Menon, 2013). Recent studies have shown that another member of large GST family, glutathione transferase omega 1 (GSTO1-1), also plays a role in the glutathionylation cycle catalyzing both the glutathionylation and deglutathionylation of proteins as a part of the redox response phenomenon. Both GSTP1 and GSTO1 are downregulated in an animal model of PC (Mavis et al., 2009); therefore, it would be interesting to study whether polymorphic expression of *GSTO1* also poses a risk of PC. Regarding genetic variations in *GSTO1*, a clear functional significance exists in terms of *GSTO1* polymorphism, causing alanine to aspartate substitution in amino acid 140 (rs4925, c.419C > A, p.Ala140Asp), which results in a change in its deglutathionylase activity (Menon and Board, 2013). In addition to *GSTO1*, another GST omega class member, *GSTO2*, also exhibits functional polymorphism that might be relevant to prostate cell redox homeostasis. GSTO2 catalyzes the regeneration of ascorbic acid, by its dehydroascorbate reductase activity. Thus, for *GSTO2* polymorphism, which causes an asparagine to aspartate substitution in amino acid 142 (rs156697, c.424A > G, p.Asn142Asp), a strong association between GSTO2*G variant allele and lower *GSTO2* gene expression has been shown (Mukherjee et al., 2006).

In the case of PC, a growing body of evidence suggests that GSTs might be involved not only in the development but also in the progression of the disease. However, only a few studies have addressed the role of GST polymorphisms with regard to the survival of PC patients (Agalliu et al., 2006; Chen et al., 2013; Acevedo et al., 2014), proposing the important catalytic and regulatory roles as the potential underlying molecular mechanisms involved in PC progression. Considering the novel pleiotropic roles of GST omega class members in redox homeostasis, we hypothesized that polymorphisms in *GSTO1* and *GSTO2* might contribute to PC development and progression. Therefore, we performed a comprehensive analysis of *GSTO1* and *GSTO2* SNPs’ role in susceptibility to PC, as well as whether they might serve as prognostic biomarkers independently or in conjunction with other common GST polymorphisms.

## Materials and Methods

### Study Group

A total of 473 participants were included in this case–control study (237 patients and 236 controls). We enrolled 237 patients from the Urology Clinic, Clinical Center of Serbia, Belgrade, and Institute for Oncology and Radiology of Serbia, Belgrade, Serbia, with histologically confirmed PC. After obtaining informed consent, data on PC diagnostics and treatment were taken from medical records and medical history, whereas a questionnaire about demographics, physical activity, and reproductive and benign prostate hyperplasia history was conducted. The control group was comprised of 236 individuals recruited from the Urology Clinic, Clinical Center of Serbia, Belgrade, with no previous personal history of malignant disease matched to PC patients according to age. Written informed consent was obtained from all participants in the study and the study protocol was approved by the Ethical Committee of the University of Belgrade, Faculty of Medicine (approval number: 2,650/IV-21, April 10, 2018). The research was carried out in compliance with the Declaration of Helsinki.

### DNA Isolation and Glutathione Transferases Genotyping

QIAamp DNA mini kit (Qiagen, Hilden, Germany) was used to isolate DNA from the whole blood of all subjects according to the manufacturer’s instructions. Determination of *GSTO1 rs4925* and *GSTO2 rs156697* was performed by quantitative polymerase chain reaction (qPCR) using TaqMan SNP Genotyping assays (Thermo Fisher Scientific, Waltham, MA, United States, assay ID: C_11,309,430_30 and C_3,223,136_1, respectively) on Mastercycler ep realplex (Eppendorf, Hamburg, Germany). Into each well of the reaction plate, 5 µL of the sample was applied and dried down at 65°C in 30 min. Then, 2.50 µL of commercial MasterMix, 0.25 µL of TaqMan probe, and 2.25 µL of DNAse-free water were mixed in a total volume of 5 µL and added to the plate. The thermal protocol for gene amplification consisted of 4 min of initial denaturation and 40 repeated cycles (15 s at 95°C and 1 min at 60°C). The genotypes were analyzed according to the Eppendorf realplex software instructions. Genotyping of *GSTM1* and *GSTT1* gene deletions, as well as GSTP1 rs1695 and GSTP1 rs1138272 polymorphisms, was performed as previously described (Santric et al., 2020).

### Statistical Analysis

Statistical Package for the Social Sciences (SPSS software version 17, SPSS Inc., Chicago, IL, United States) was used for statistical analysis. Multinominal logistic regression was used for calculating odds ratio (OR) and 95% confidence interval (95%CI) in order to determine the potential association between *GSTO1* and *GSTO2* genotypes and risk for the development of PC. Age, presence of diabetes mellitus type 2, and hypertension were considered as confounding factors in analysis. The linkage disequilibrium (LD) between SNPs and haplotype analysis was executed by SNPStats software available online (Solé et al., 2006). Patients were followed for a maximum of 50 months (from January 2014 to March 2018) until death or the end of the follow-up period. The median time of follow-up was 42 months (range 1–50 months). During this time, three patients were lost. Kaplan-Meier method was used for calculating mean survival time and computing survival curves. Evaluation of variation in survival time between different GST genotypes was calculated with the log-rank test. Cox proportional hazard regression models were calculated to assess the predictive value of GST genotypes in overall mortality in two models, unadjusted and adjusted for the presence of diabetes mellitus type 2 which was the only confounding factor in the study group that was independently associated with worse outcome. Results were considered statistically significant if *p*-value was ≤ 0.05.

## Results

Demographic and clinical characteristics of PC patients (*n* = 237) and controls (*n* = 236) are shown in Table 1. There was no statistically significant difference in age, body mass index, and smoking habits (*p* > 0.05), whereas the presence of diabetes and hypertension was significantly higher in the patient in comparison to the control group (*p* < 0.001 and *p* = 0.002, respectively). As presented in Table 1, the majority of PC patients had prostate-specific antigen (PSA) > 20 ng/ml at the time of diagnosis (35%), while Gleason score was 7 (3 + 4) (27%). During the median follow-up period of 42 months ranging from 1 to 50 months, 23 patients died of PC and three were lost to follow-up.

**TABLE 1:** Selected demographic and clinical characteristics of subjects.

Characteristics	Controls, *n* (%)	Patients, *n* (%)			p** value
		PC deaths	Alive	p** value	
Age,* years	67.35 ± 9.18	71.32 ± 7.03	68.55 ± 6.87	0.074	0.052
Time to event (months)^a^		26.23 ± 13.26	42.44 ± 3.23	< 0.001	
BMI (kg/m^2^)^b^					
< 25	57 (33)	7 (32)	64 (30)	0.906	0.119
25–29.9	95 (55)	10 (45)	105 (50)		
≥ 30	21 (12)	5 (23)	41 (20)		
Hypertension^c^					
Yes	87 (39)	14 (67)	111 (57)	0.377	< 0.001
No	135 (61)	7 (33)	85 (43)		
Diabetes mellitus type 2^d^					
Yes	12 (7)	7 (32)	30 (15)	0.041	0.002
No	174 (93)	15 (68)	173 (85)		
Smoking status^e^					
Current smoker	104 (46)	9 (43)	102 (49)	0.603	0.570
Nonsmoker	124 (54)	12 (57)	107 (51)		
PSA at diagnosis (ng/ml)					
< 10		8 (35)	70 (33)	0.639	
10–20		8 (35)	61 (29)		
> 20		6 (26)	76 (35)		
Missing/unknown		1 (4)	7 (3)		
Gleason score					
≤ 6		4 (17)	52 (24)	0.703	
7 (3 + 4)		5 (22)	57 (27)		
7 (4 + 3)		3 (13)	35 (16)		
8		1 (4)	27 (13)		
9/10		3 (13)	19 (9)		
Missing/unknown		7 (31)	24 (11)		

^*^Mean value ± standard deviation; **p-value of Pearson’s chi-square or Fisher’s exact test for categorical variables and *t*-test for two independent samples for continuous variables; p ≤ 0.05 was considered to be statistically significant.

^a^ Time in months elapsed from the date of inclusion in the study until the date of prostate cancer death (*n* = 23) or end of follow-up (*n* = 214) written as mean value ± standard deviation.

^b^Information was accessible for 173 of 236 controls and 232 of 237 patients.

^c^Information was obtainable for 222 of 236 controls and 217 of 237 patients.

^d^ Information was accessible for 186 of 236 controls and 228 of 237 patients.

^e^ Current smokers included patients who declared themselves as smokers at the time of recruitment to study; information was accessible for 228 of 236 controls and 230 of 237 patients.

The distribution of the *GSTO1 rs4925* and *GSTO2 rs156697* genotypes and alleles is shown in Table 2. As presented, the carriers of GSTO1*A/A variant genotype were at a 2.1-fold higher risk of developing PC compared to carriers of referent GSTO1*C/C genotype (*p* = 0.033). The logistic regression analysis of *GSTO2 rs156697* polymorphism showed that heterozygous genotype GSTO2*A/G carries a 1.6-fold increased risk for PC development in comparison with referent GSTO2*A/A genotype (*p* = 0.041). The risk slightly increased when carriers of either GSTO2*A/G or GSTO2*G/G genotype were compared to the carriers of referent genotype (OR = 1.75, 95%CI: 1.14–2.68, *p* = 0.010). The highest risk of PC was shown for GSTO2*G/G variant genotype (OR = 2.55, 95%CI: 1.28–5.08, *p* = 0.008). At the allelic level, GSTO2*G held a 1.48-fold higher risk for disease development than GSTO2*A allele (*p* = 0.005). When *GSTO1 rs4925* and *GSTO2 rs156697* polymorphisms were analyzed in combination, carriers of at least one variant GSTO1*A and GSTO2*G alleles had a 1.8-fold higher risk of developing PC compared to referent genotype combination (95%CI: 1.07–2.96, *p* = 0.026) (Table 2).

**TABLE 2:** Distribution of individual *GSTO1 rs4925* and *GSTO2 rs156697* genotypes and alleles, as well as combined *GSTO1*/*GSTO2* genotypes in controls and PC patients.

Genotype	Controls, *n (%)*	Patients, *n (%)*	OR (95% CI)^a^	p* value
***GSTO1 rs4925*** **^b^**				
*C/C	82 (37)	68 (31)	1.00	
*C/A	115 (52)	116 (53)	1.34 (0.85–2.13)	0.210
*A/A	23 (11)	36 (16)	2.12 (1.06–4.23)	0.033
*C/C	82 (37)	68 (31)	1.00	
*CA+*AA	138 (63)	152 (69)	1.47 (0.94–2.28)	0.090
*C	277 (63)	254 (57)	1.00	
*A	161 (37)	188 (43)	1.27 (0.97–1.67)	0.080
***GSTO2 rs156697*** **^c^**				
*A/A	95 (45)	74 (32)	1.00	
*A/G	97 (46)	119 (52)	1.59 (1.02–2.49)	0.041
*G/G	20 (9)	36 (16)	2.55 (1.28–5.08)	0.008
*A/A	95 (45)	74 (32)	1.00	
*AG+*GG	117 (55)	155 (68)	1.75 (1.14–2.68)	0.010
*A	285 (68)	269 (58)	1.00	
*G	137 (32)	191 (42)	1.48 (1.12–1.94)	0.005
**Combined *GSTO1 rs4925/GSTO2 rs156697***				
*CC/*AA	57 (28)	49 (23)	1.00	
*CC/*AG + *GG	20 (10)	18 (8)	1.30 (0.55–3.05)	0.553
*CA+*AA/*AA	35 (17)	20 (9)	0.87 (0.41–1.83)	0.715
*CA+*AA/*AG+*GG	92 (45)	129 (60)	1.78 (1.07–2.96)	0.026

*p ≤ 0.05 was considered to be statistically significant.

^a^OR: odds ratio adjusted to age, hypertension, and diabetes mellitus type 2 for genotypes and unadjusted for alleles; 95% CI: 95% confidence interval.

^b^ For *GSTO1 rs4925*, genotyping was successful in 220 of 236 controls’ and 220 of 237 patients’ samples.

^c^ For *GSTO2 rs156697*, genotyping was successful in 212 of 236 controls’ and 229 of 237 patients’ samples.

The results on individual and combined effects of *GSTO* polymorphisms obtained by logistic regression analysis were also confirmed by haplotype analysis, performed in accordance to linkage disequilibrium (LD) found between these SNPs (D′ = 0.66, *p* < 0.001). The most frequent haplotype among controls (55%) and patients (50%) was H1, represented by GSTO1*C and GSTO2*A referent alleles. Carriers of H2 haplotype, consisting of both GSTO1*A and GSTO2*G variant alleles, had significantly increased risk of PC (OR = 1.59, 95%CI: 1.12–2.25, *p* = 0.009) (Table 3).

**TABLE 3:** Haplotypes of *GSTO1 rs4925* and *GSTO2 rs156697* in relation to the risk of prostate cancer.

Haplotype	*GSTO1 rs4925*	*GSTO2 rs156697*	Controls, %	Patients, %	OR (95% CI)^a^	p* value
H1	*C	*A	55	50	1.00	
H2	*A	*G	25	34	1.59 (1.12–2.25)	0.009
H3	*A	*A	12	9	0.98 (0.55–1.74)	0.940
H4	*C	*G	8	7	1.41 (0.72–2.77)	0.320

Global haplotype association *p*-value: 0.045; *p ≤ 0.05 was considered to be statistically significant.

^a^OR: odds ratio adjusted to age, hypertension, and diabetes mellitus type 2; 95% CI: 95% confidence interval.

**FIGURE 1:**
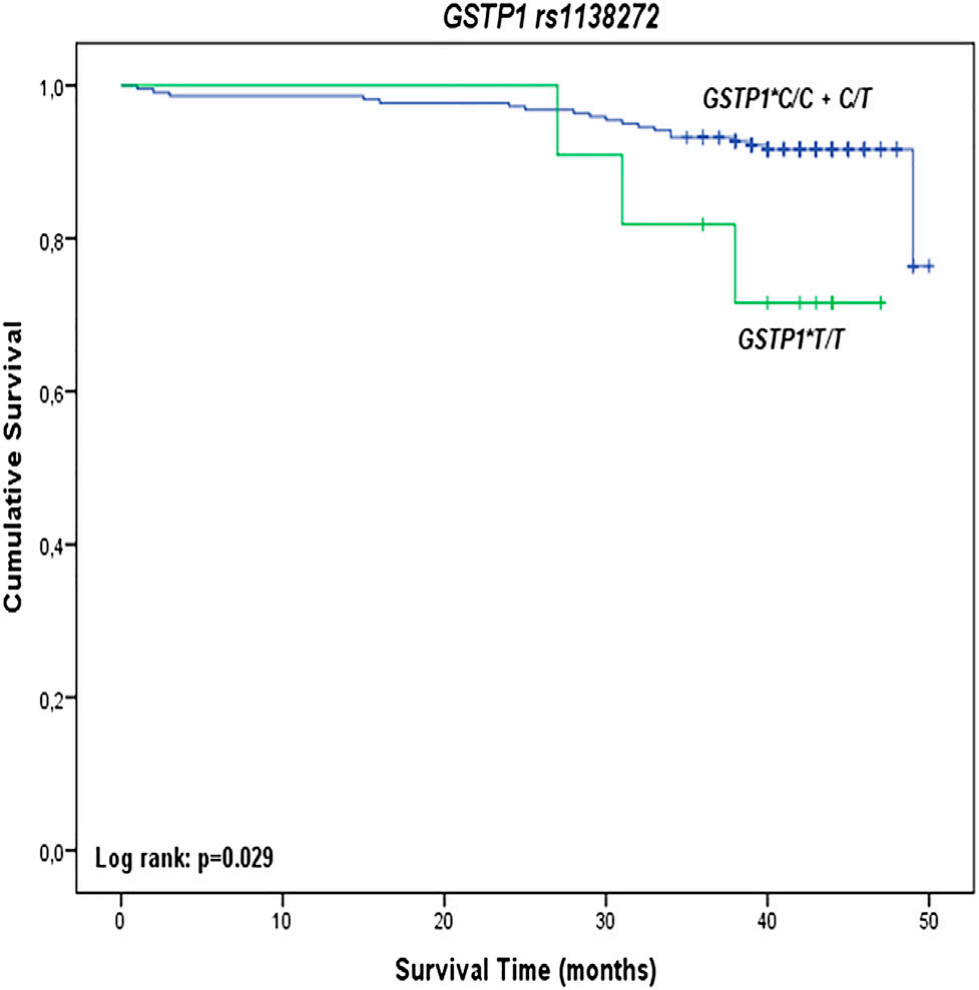
Overall survival of PC patients stratified by *GSTP1 rs1138272* polymorphism.

Kaplan-Meier survival analysis showed a statistically significant effect of *GSTP1 rs1138272* polymorphism on overall survival among PC patients (Figure 1). Namely, carriers of GSTP1*T/T variant genotype had shorter overall survival (log-rank: *p* = 0.029) compared to carriers of at least one GSTP1*C referent allele (Figure 1). However, *GSTM1*, *GSTT1*, *GSTO1*, *GSTO2,* and *GSTP1* rs1695 polymorphisms did not show effect on overall survival among PC patients (Supplementary Figure S1).

The multivariate Cox regression analysis confirmed GSTP1*T/T genotype as an independent predictor of higher risk for overall mortality in PC patients (Table 4). Namely, carriers of GSTP1*T/T variant genotype had a 3.6-fold higher mortality risk compared to the homozygous carriers of GSTP1*C allele in Model 1 (HR = 3.65, 95%CI: 1.02–13.12, *p* = 0.047). In Model 2, adjusted for the presence of diabetes mellitus type 2, mortality risk was increased to more than 4-fold (HR = 4.67, 95%CI: 1.26–17.37, *p* = 0.021). The multivariate Cox regression analysis did not show a statistically significant association between investigated *GSTM1*, *GSTT1*, *GSTO1*, *GSTO2*, and *GSTP1* rs1695 polymorphisms and overall mortality.

**TABLE 4:** Gene polymorphisms of *GSTM1*, *GSTT1*, *GSTO1 rs4925*, *GSTO2 rs156697*, *GSTP1 rs1695*, and *GSTP1 rs1138272* and risk of overall mortality in prostate cancer patients by Cox proportional hazards regression models.

Genotype	Model 1^a^		Model 2^b^	
	**HR (95% CI)** ^c^	p* value	**HR (95% CI)**	***P* value**
***GSTM1***				
active	1.00		1.00	
null	0.98 (0.42–2.27)	0.957	0.99 (0.42–2.34)	0.983
***GSTT1***				
active	1.00		1.00	
null	1.53 (0.68–3.47)	0.307	1.51 (0.65–3.52)	0.335
***GSTO1 rs4925***				
*C/C	1.00		1.00	
*C/A	0.99 (0.37–2.71)	0.997	1.13 (0.39–3.29)	0.818
*A/A	1.39 (0.39–4.94)	0.609	1.76 (0.47–6.59)	0.403
***GSTO2 rs156697***				
*A/A	1.00		1.00	
*A/G	0.83 (0.33–2.06)	0.680	0.94 (0.37–2.44)	0.906
*G/G	0.77 (0.20–2.89)	0.695	0.86 (0.22–3.33)	0.828
***GSTP1 rs1695***				
*A/A	1.00		1.00	
*A/G	0.69 (0.27–1.74)	0.430	0.77 (0.30–1.94)	0.575
*G/G	1.02 (0.34–3.07)	0.967	0.80 (0.25–2.63)	0.718
***GSTP1 rs1138272***				
*C/C	1.00		1.00	
*C/T	1.05 (0.42–2.63)	0.917	1.36 (0.53–3.50)	0.524
*T/T	3.65 (1.02–13.12)	0.047	4.67 (1.26–17.37)	0.021

*p ≤ 0.05 was considered to be statistically significant.

^a^ Model 1 represents unadjusted results.

^b^ Model 2 was adjusted for the presence of diabetes mellitus type 2.

^c^HR: hazard ratio; 95% CI: 95% confidence interval.

## Discussion

The results of this study, for the first time, demonstrated that homozygous carriers of both GSTO1*A/A and GSTO2*G/G variant genotypes are at increased risk of PC. This was further confirmed by haplotype analysis which showed that H2 comprised of both GSTO1*A and GSTO2*G variant alleles represented a high-risk combination. However, prognostic relevance of polymorphisms in GST omega genes was not found in our cohort of patients with PC. Analysis of the role of other investigated GSTs polymorphisms (*GSTM1*, *GSTT1*, and *GSTP1*) in terms of PC prognosis has shown statistically significant shorter survival in carriers of GSTP1*T/T (*rs1138272*) genotype compared to those carrying at least one referent allele. In addition, carriers of GSTP1*T/T genotype independently predicted a four-fold higher risk of overall mortality among these patients.

As a result of functional polymorphism, GSTO1*C referent allele exhibits high deglutathionylase activity and minor activity in the forward glutathionylation reaction in contrast to GSTO1*A variant allele (Menon and Board, 2013). Our results showed that homozygous carriers of GSTO1*A/A variant genotypes are at increased risk of PC, which is in agreement with another urological cancer, clear cell renal cell carcinoma. Our data on the association of GSTO2*G variant allele with increased risk of PC are in line with previous findings on ovarian (Pongstaporn et al., 2006), breast (Xu et al., 2014), bladder (Djukic et al., 2015), and clear cell renal cell carcinoma (Radic et al., 2018). Moreover, as a result of LD found between these SNPs, carriers of H2 haplotype, consisting of both GSTO1*A and GSTO2*G variant alleles, had significantly increased risk of PC. It seems that these enzymes are involved in the regulation of redox homeostasis in a synergistic way, affecting the susceptibility of PC by several mechanisms. In view of the fact that glutathionylation, as a posttranslational modification, can affect the activity of many proteins involved in tumor growth, while the deglutathionylation process also may expose vulnerable thiol groups in prostate tissue to oxidation, the role of *GSTO1* and *GSTO2* allelic variants exhibiting altered activities could provide a plausible mechanism to explain the associations between these genetic polymorphisms and risk for PC development.

In the course of PC carcinogenesis, one of the initial molecular alterations is the activation of the proto-oncogene c-myc, followed by overexpression of hypoxia-inducible factor-1α (HIF-1α) (Boldrini et al., 2019). As a central player of intratumoral hypoxia, HIF-1α has been associated with shorter time to biochemical recurrence, metastasis, and chemoresistance in PC patients, being an attractive target for cancer therapy (Vergis et al., 2008; Ranasinghe et al., 2013). Interestingly, among the complex changes of cellular redox regulation induced by intratumoral hypoxia, increase in the S-glutathionylation of HIF-1α and its expression in colon cancer cells has been demonstrated recently (Jeon et al., 2018). Moreover, by inducing expression of the major intracellular enzyme with deglutathionylase activity, glutaredoxin 1, Jeon and coworkers confirmed the relevance of HIF-1α glutathionylation for its activity in colon cancer cells (Jeon et al., 2018). Under hypoxic conditions, expression of HIF-1α was not induced in these glutaredoxin 1-overexpressing cells. It is questionable whether GSTO1*A variant allele with lower deglutathionylase activity contributes to stabilization in HIF-1α in the glutathionylated state. In addition to regulation by glutathionylation, inefficient regeneration of ascorbic acid, as a possible consequence of *GSTO2* polymorphism, might affect HIF signaling pathway in a different way. Namely, oxygen-dependent protein hydroxylases, which are mediators of ubiquitinylation-proteasomal degradation of HIF, are dependent on vitamin C as a cofactor. It might be speculated that vitamin C-dependent inhibition of the HIF pathway may provide an additional approach for controlling tumor progression (Li and Schellhorn, 2007).

Regarding the prognostic role of GSTs in PC, potential biomarker relevance was demonstrated for *GSTM1*, *GSTT1*, and *GSTP1 rs1695* polymorphisms (Agalliu et al., 2006; Chen et al., 2013; Cotignola et al., 2013; Acevedo et al., 2014). Indeed, *GSTM1* deletion polymorphism has been suggested as a useful biomarker to identify patients at higher risk for death from PC (Agalliu et al., 2006), while *GSTM1-active* and *GSTT1-null* genotypes seem to be good prognostic markers, especially in patients with advanced tumors (Acevedo et al., 2014). Regarding two commonly occurring *GSTP1* polymorphisms (rs1695 c.313A > G, p.IIe105Val and rs1138272 c.341C > T, p.Ala114Val), only *GSTP1 rs1695* polymorphism was analyzed and showed no prognostic significance, which is in line with our results on this polymorphism (Cowell et al., 1988; Wei et al., 2013; Zhang et al., 2016). Still, the implementation of *GSTP1* genotyping has been suggested as a novel biomarker to identify patients at risk of recurrence, as well as personalized therapies in PC management (Cotignola et al., 2013). Interestingly, in our study, for the first time, we found that GSTP1*T/T *(rs1138272)* genotype has a significant prognostic value.

Recently, it has been shown that neoplastic transformation by silencing *GSTP1* tumor suppressor gene function might be mediated by activation of c-myc (Boldrini et al., 2019), whereas the significant protective effect was shown after GSTP1 overexpression in PC *in vitro* and *in vivo* (Wang et al., 2017). Besides, the protective effect of *GSTP1* haplotype comprised of two connected *GSTP1* SNPs was associated with more efficient protection against carcinogenic compounds in PC susceptibility, as suggested recently (Santric et al., 2020). Namely, the results of our previous study have shown that carriers with at least one copy of the GSTP1*T (*rs1138272*) or GSTP1*G (*rs1695*) variant allele are at a significantly higher risk of PC development. The effect on PC susceptibility was even more pronounced when both GSTP1 variant alleles were present in combination (Santric et al., 2020). In this line, the results from this study indicated that carriers of GSTP1*T/T genotype independently predicted a four-fold higher risk of death among these patients. It is important to note that, in addition to its catalytic role in conjugation of various electrophilic compounds including chemotherapeutic agents, GSTP1 also participates in the process of glutathionylation, as well as the regulation of redox-dependent apoptotic signaling (Pljesa-Ercegovac et al., 2018). The importance of glutathionylation status and redox disturbance during PC progression was also suggested in the modulation of the transcriptional activity of androgen (AR) and estrogen receptor (ER) α (Bonkhoff et al., 1999; Shiota et al., 2011; Xiong et al., 2012; Zhang et al., 2018). Thus, oxidative stress has recently been suggested to convert androgen-dependent PC into castration-resistant PC through various mechanisms, including glutathionylation of ERα.

This study has some limitations that need to be addressed. This case–control study included only population of Serbian males; therefore, these results should be carefully interpreted in general population. Validation of our results would require a larger sample size that would include men of different ethnicities and races.

In conclusion, the role of GSTO and GSTP class in redox regulation, especially glutathionylation/deglutathionylation cycle, might be suggested as a potential mechanism in the process of PC adaptation to oxidative stress. Our data on the effects of both GSTO1*A/A and GSTO2*G/G variant genotypes on the risk of PC have the potential to improve the susceptibility biomarkers development in the field of urologic oncology. Besides the results on the effect of novel *GSTP1* polymorphism on the overall survival of patients with PC, it would be beneficial to investigate its potential association with cancer-specific survival in a larger cohort. Moreover, future functional investigation and interconnection of *GSTP1* polymorphisms and HIF-1α regulation could provide better outcomes and therapeutic chances for men with PC.

## Data Availability

The original contributions presented in the study are included in the article and Supplementary Material, further inquiries can be directed to the corresponding authors.

## Abbreviations

GST, glutathione-S transferases; GSTO, glutathione-S transferase omega 1; GSTO2, glutathione-S transferase omega 2; PC, prostate cancer; GSTM1, glutathione-S transferase mu 1; GSTT1, glutathione-S transferase theta 1; GSTP1, glutathione-S transferase pi 1; PCR, polymerase chain reaction; SNP, single-nucleotide polymorphism; PSA, prostate-specific antigen; HIF1-1α, hypoxia-inducible factor-1α; AR, androgen receptor; ERα, estrogen receptor α.
