# Supplementary figures and images for: Polymorphisms in Genes Encoding Glutathione Transferase Pi and Glutathione Transferase Omega Influence Prostate Cancer Risk and Prognosis
Source: Front Mol Biosci. 2021 Apr 14;8:620690. doi: 10.3389/fmolb.2021.620690 (PMC8079946; doi:10.3389/fmolb.2021.620690)

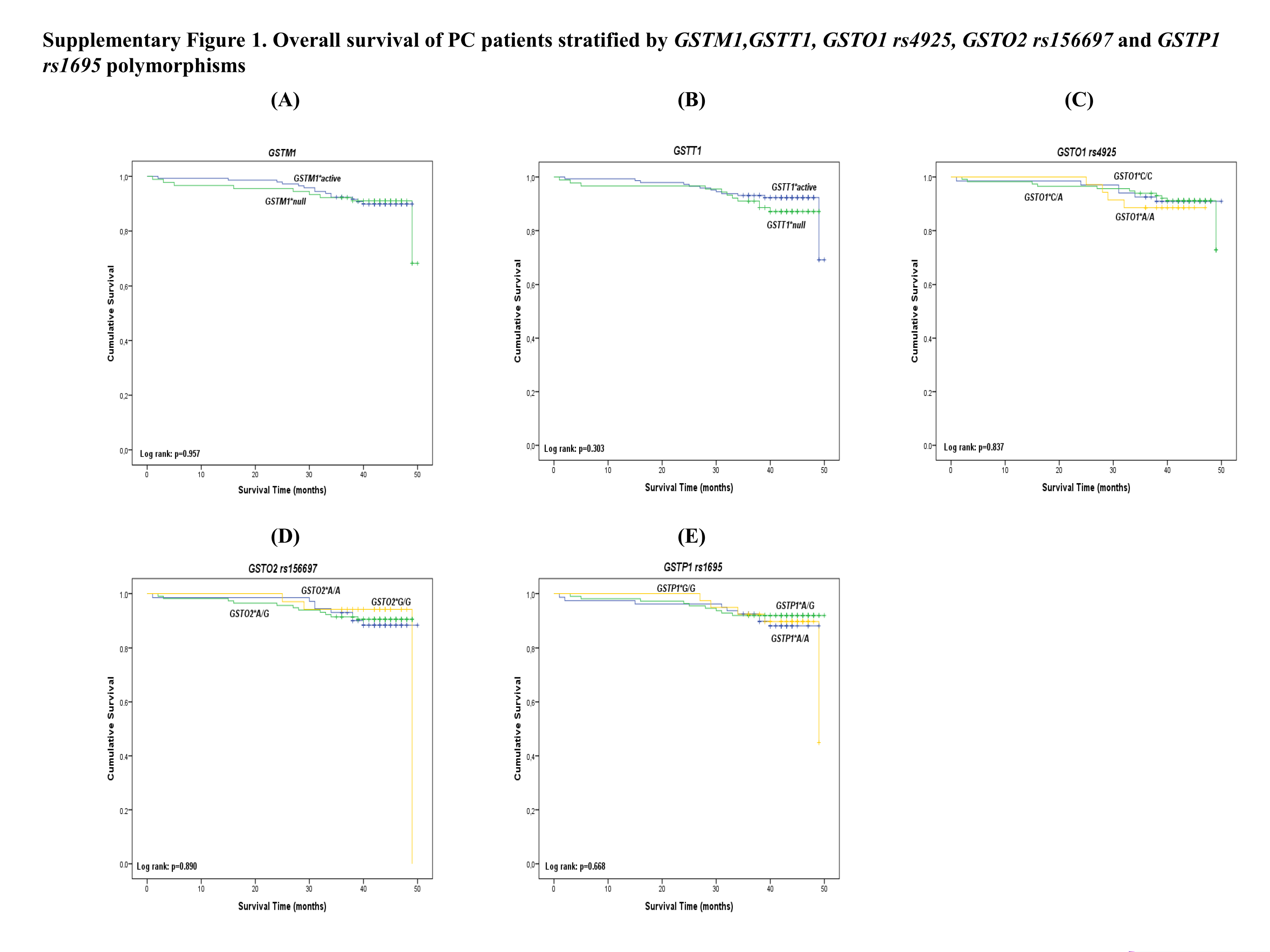

Supplement: Supplementary file 1 [file image1.tif]
